# Fc Binding by FcγRIIa Is Essential for Cellular Activation by the Anti-FcγRIIa mAbs 8.26 and 8.2

**DOI:** 10.3389/fimmu.2021.666813

**Published:** 2021-10-25

**Authors:** Bruce D. Wines, Halina M. Trist, Sandra Esparon, Rachael E. Impey, Graham A. Mackay, Robert K. Andrews, Tatiana P. Soares da Costa, Geoffrey A. Pietersz, Ross I. Baker, P. Mark Hogarth

**Affiliations:** ^1^ Immune Therapies Laboratory, Burnet Institute, Melbourne, VIC, Australia; ^2^ Department of Immunology and Pathology, Central Clinical School, Monash University, Melbourne, VIC, Australia; ^3^ Department of Clinical Pathology, The University of Melbourne, Parkville, VIC, Australia; ^4^ Department of Biochemistry and Genetics, La Trobe Institute for Molecular Science, La Trobe University, Melbourne, VIC, Australia; ^5^ Department of Biochemistry and Pharmacology, The University of Melbourne, Parkville, VIC, Australia; ^6^ Department Cancer Biology and Therapeutics, John Curtin School of Medical Research, The Australian National University, Canberra, ACT, Australia; ^7^ Atherothrombosis and Vascular Biology Laboratory, Baker Heart and Diabetes Institute, Melbourne, VIC, Australia; ^8^ Perth Blood Institute, Murdoch University, Perth, WA, Australia; ^9^ Western Australian Centre for Thrombosis and Haemostasis, Murdoch University, Murdoch, WA, Australia

**Keywords:** Fc receptor, IgG, FcγRIIa, effector function, antibody dependent cellular cytotoxicity (ADCC), mAb - monoclonal antibody

## Abstract

FcγR activity underpins the role of antibodies in both protective immunity and auto-immunity and importantly, the therapeutic activity of many monoclonal antibody therapies. Some monoclonal anti-FcγR antibodies activate their receptors, but the properties required for cell activation are not well defined. Here we examined activation of the most widely expressed human FcγR; FcγRIIa, by two non-blocking, mAbs, 8.26 and 8.2. Crosslinking of FcγRIIa by the mAb F(ab’)_2_ regions alone was insufficient for activation, indicating activation also required receptor engagement by the Fc region. Similarly, when mutant receptors were inactivated in the Fc binding site, so that intact mAb was only able to engage receptors *via* its two Fab regions, again activation did not occur. Mutation of FcγRIIa in the epitope recognized by the agonist mAbs, completely abrogated the activity of mAb 8.26, but mAb 8.2 activity was only partially inhibited indicating differences in receptor recognition by these mAbs. FcγRIIa inactivated in the Fc binding site was next co-expressed with the FcγRIIa mutated in the epitope recognized by the Fab so that each mAb 8.26 molecule can contribute only three interactions, each with separate receptors, one *via* the Fc and two *via* the Fab regions. When the Fab and Fc binding were thus segregated onto different receptor molecules receptor activation by intact mAb did not occur. Thus, receptor activation requires mAb 8.26 Fab and Fc interaction simultaneously with the same receptor molecules. Establishing the molecular nature of FcγR engagement required for cell activation may inform the optimal design of therapeutic mAbs.

## Introduction

IgG Abs, elicited by vaccination or natural infection or as therapeutic mAbs, are important mediators of human health and the activation of FcγR-expressing innate leucocytes is often fundamental to their efficacy. IgG antibodies have proved themselves highly useful therapeutics, a trend that continues with almost 90 new IgG therapeutics reported in late-stage clinical trials in 2020 (1). The family of FcγRs vary in their affinity for ligand, their structures and organization, tissue expression and the types of functions they trigger, altogether equating to diverse roles in normal immunity (2–8). FcγRIIa binds monomeric IgG with low (~10^6^ M^-1^) affinity (9, 10) and activates cells only by avid interaction with IgG immune complexes (IC) or IgG opsonized targets (11). The activating FcγRs, including FcγRIIa, signal *via* an immunoreceptor tyrosine-based activation motif (ITAM) (12), with FcγRIIa and FcγRIIc containing their ITAM within their cytoplasmic domains, while other activating FcRs associate with an ITAM containing signal transduction subunit, FcRγ (13, 14). FcγRIIa has two major polymorphisms, H131, the only functional FcγR for human IgG2, and R131 which was characterized functionally by its preferential interaction with mouse IgG1 (15, 16). FcR activation occurs upon receptor clustering leading first to trans-phosphorylation of the cytoplasmic domains of receptors and/or their subunits by a pre-associated src-family kinase (17). Depending on the effector cell stimulated, cellular responses to FcγRIIa activation may include, phagocytosis, respiratory burst, degranulation, cytokine production, mediator release and antigen presentation (2–8). FcγRIIa is the most widely expressed FcγR of human leukocytes and the only FcγR on platelets (18). FcγRIIa potently activates platelets when engaged by the Fc of anti-platelet antibodies (19, 20), including anti-platelet factor 4 (PF4) antibodies which trigger heparin induced thrombocytopenia (21, 22). FcγRIIa also triggers a rare thrombotic thrombocytopenia induced by anti-PF4 antibodies after vaccination for SARS CoV-2 spike vectored by a recombinant adenovirus (ChAdOx1, AstraZeneca) (23, 24).

The molecular basis for ligand (IgG) recognition by FcγRs (25–28), including FcγRIIa (29), has been well defined by structural studies. However, despite the importance of this event and its intensive structural investigation, relatively little is known of the requirements for the engagement of FcγRs for optimal cell activation (30). Some mAbs can themselves also activate FcγRIIa although the properties of these mAbs are not defined. We previously used two agonistic and non-blocking anti-FcγRIIa mAbs, mAb 8.26 and mAb 8.2 to explore FcγRIIa activation (31). These non-blocking anti-FcγRIIa mAbs activate FcγRIIa and mAb 8.2 has been used in a number of studies (32, 33) including high resolution SIM (structural illuminated microscopy) that found mAb 8.2 treatment drives FcγRIIa into aggregates on the cell surface (32). Using defined ligands and mutants of FcγRIIa we now investigate the essential features of FcγRIIa engagement by these mAbs that are necessary for receptor signaling and cell activation.

## Materials and Methods

### MAbs and Reagents

The human FcγRIIa specific murine mAbs 8.2 (IgG1), 8.7 (IgG1), 8.26 (IgG2b) (31), IgG1 isotype control mAb X68 (34) and the production of F(ab’)_2_ regions (31) are as described previously. Albumin, bovine Fraction V and other fine chemicals were from Sigma-Aldrich. Stain-free SDS-PAGE gels and PVDF transfer membrane were from BioRad Laboratories (Melbourne, Australia) and 3,3′,5,5′-tetramethylbenzidine (TMB) ELISA substrate was from Life Technologies (Thermo Fisher, Melbourne, Australia).

### FcγRIIa and Recombinant Ab Expression

The expression vectors for unmodified wild-type (WT) and mutant chimeric (murine V domain-anti-TNP/human constant) IgG are as described previously (35, 36). Recombinant anti-TNP heavy WT and double mutant S267E, L328F IgG1 (SELF) (37) and light chains in pCR3 (Invitrogen) were expressed by co-transfection in Expi293F™ cells (Gibco Life Technologies) according to the manufacturer’s instructions. The antibodies were purified by protein A chromatography followed by gel filtration on a Superose 6 column performed using an AKTA Purifier (GE Healthcare Life Sciences, Melbourne, Australia) (35).

WT and mutant FcγRIIa expression used cDNA [FcγRIIa; clone Hu3.0 (38)] templates and PCR reactions were performed using mutagenic primers and polymerases *Pwo* (Roche) or AccuPrime™ *Pfx* (Invitrogen, Life Technologies). Other standard DNA manipulation used enzymes from New England Biolabs. Sequencing and expression of FcγRIIa pMX based vectors in IIA1.6 cells (39) and rsFcγRIIa-hexahistidine-AviTag-biotin (36) were as described previously.

### FcγRIIa Phosphorylation ELISA

FcγRIIa capture used mAb 8.7 or mAb IV-3 coated at 20 µg/ml to F96 Maxisorp plates (Nunc) which were then blocked with PBS containing 1% BSA to each well (130 µl). The epitopes of the mAbs 8.7 and IV−3 (29, 31) differ from that of mAbs 8.2 and 8.26 defined in this study. IIA1.6 cells expressing the appropriate receptor were cultured in RPMI-1640 or DMEM medium supplemented with 5% fetal bovine serum and 2 mM glutamine, added to a round bottom plate (2x10^5^ cells/well; >95% viable), plates were centrifuged (1000 rpm; 5 min) and most of the media removed by a single rapid inversion of the plate. Plates were warmed on an aluminum-foil mat heating block (37°C; 2 min). Receptor stimulation was initiated by adding 20 µl of agonist in tissue culture media. At the indicated times, reactions were stopped by adding lysis buffer (100 ul; 20 mMTris, 150 mM NaCl, 2 mM NaF pH7.4 containing 1% Brij96, 1 mM activated Na_3_VO_4_ and a protease inhibitor cocktail without EDTA (Sigma Aldrich/Roche)). Lysates were then transferred to the mAb 8.7 or mAb IV-3 coated capture plate and incubated overnight at 4°C or 2h at 37°C. Plates were emptied by inversion and filled with 130 µl wash buffer (220 mM Tris, 150 mM NaCl, 2 mM NaF pH7.4, 0.05% Tween 20) supplemented with activated Na_3_VO_4_ (1 mM) emptied and 2 µg/ml biotinylated anti-phosphotyrosine mAb 4G10 (4G10-biotin) in wash buffer containing 1 mM activated Na_3_VO_4_ and 1% BSA added and incubated for 2h at 25°C. Plates were emptied by inversion and filled with 130 µl wash buffer supplemented with 1 mM activated Na_3_VO_4_, emptied and incubated with a 1/6000 dilution of streptavidin-HRP conjugate (Cytiva/Amersham) for 1h at 25°C. Unbound conjugate was removed by 4 cycles of emptying the wells and refilling with wash buffer and emptying again. The bound conjugate was detected by incubation with TMB substrate (typically 10 min), stopping with 1M HCl and recording the absorbance at 450 nm. Phosphorylation curves were constructed with subtraction of the zero-time point, which was typically an A450nm < 0.05 after subtraction of the reagent blank.

### Platelet Activation Assay

Platelet rich plasma (PRP) was prepared by collecting whole blood into 3.2% (w/v) trisodium citrate and removing the cellular components by centrifugation (120g, 20 min, all steps 22°C). Activation was performed by incubating 20 µl PRP with 100 µl of 10 mM Tris, 150 mM NaCl pH 7.4 containing mAb, whole or F(ab’)_2_ for 30 min, or with the control platelet activator ADP (20 µM; 10 min). Staining was performed without washing, using 20 µl of mouse anti-P-selectin (AK-4) phycoerythrin-conjugate or mouse anti-human CD41a phycoerythrin-conjugate (HIP8; BD Pharmingen, 30 min). Stained platelets were collected by centrifugation (2 min, 300g) and resuspended in 10 mM Tris, 150 mM NaCl, 5 mM EDTA pH 7.4 two times. Platelets were visualized by flow cytometry with a forward and side scatter gate confirmed by CD41a staining.

### Immunoprecipitation, Electrophoresis, and Western Blotting

MAbs 8.7 (IgG1) F(ab’)_2_ and intact IV-3 were coupled to cyanogen bromide activated Sepharose according to the manufacturer’s instructions (GE Life sciences). IIA1.6 cells expressing FcγRIIa were cultured in RPMI-1640 or DMEM medium as above. Cells were adjusted to 4x10^6^ cells/40 µl and at the indicated times, 20 µl of the agonist mAbs (1-20 µg/ml in RPMI-1640 culture medium) were added. Reactions were terminated by the addition of 200 µl of lysis buffer (as above), incubated on ice (15 min), and then clarified by centrifugation (10000 rpm, 10 min at 4°C). An aliquot (10 µl) of clarified lysate was reserved for analysis and the remainder was incubated with 20 µl of mAb 8.7 F(ab’)_2_ coupled beads, 16h at 4°C. Beads were collected by centrifugation (5000 rpm, 10s) and resuspended in wash buffer. After 4 rounds of collection and resuspension in wash buffer the FcγR immune precipitates or 10 µl whole cell lysate samples were resolved on 5-15% polyacrylamide gels stain free gels (BioRad Laboratories) by SDS-PAGE and transferred to PVDF membranes. Membranes were probed with 4G10-biotin/streptavidin-HRP to detect phospho-proteins, treated with 0.02% sodium azide to quench the HRP and washed, before re-probing with rabbit anti-Syk (N19, Santa Cruz), or anti-FcγRIIa ectodomain specific rabbit anti-serum/anti-rabbit IgG-HRP.

### Flow Cytometry

MAbs and F(ab’)_2_ fragments were biotinylated using EZ-link biotinylation reagent (Pierce) according to the manufacturer’s instructions. Cells (5x10^4^) were incubated with 2 µg/ml mAbs or F(ab’)_2_ in FACS buffer (Hanks balanced salt solution containing 1mM glucose and 0.1% BSA), on ice for 30 min. Wells or tubes were filled with FACS buffer and cells collected by centrifugation, (1000rpm, 4°C, 5 min) and resuspended in 1/400 dilution of APC or PE conjugated streptavidin. Following incubation on ice for 30 min, wells or tubes were filled with FACS buffer and cells collected by centrifugation, 1000rpm, 4°C, 5 min and resuspended in FACS buffer for analysis using a Canto II flow cytometer (Becton Dickinson). Two methods for the measurement of FcR ligand binding activity were used, first TNP-BSA (~5 TNP groups per BSA) in 100 mM Na_2_HCO_3_ was labelled with Alexa-647 according to the manufacturer’s instructions and extensively dialyzed against PBS. Alexa 647-labelled TNP-BSA (1 µg/ml) was reacted with 1-10 µg/ml chimeric anti-TNP (human IgG1 constant domains) described previously (40). Second, crosslinked and biotinylated IgG was used as IC as described in (41).

The binding of anti-TNP chimeric IgG heavy chain double mutant S267E, L328F (IgG1-SELF) was detected with APC-labelled AffiniPure F(ab’)_2_ fragment of goat anti-human IgG-F(ab’)_2_ fragment specific, (Jackson ImmunoResearch Laboratories Inc., Baltimore, USA).

### Bio-Layer Interferometry

The apparent affinity of the intact mAbs and their F(ab’)_2_ fragments for FcγRIIa was measured using an Octet RED96e (FortéBio). RsFcγRIIa-WT or rsFcγRIIa-RA, with c-terminal biotin was immobilized using streptavidin (SA) biosensor tips (FortéBio) in PBS pH 7.4 containing 0.1% (w/v) BSA and 0.05% (v/v) TWEEN-20) at 25°C. Kinetic measurements made by submerging the sensors in two-fold dilution series of the mAbs or their F(ab’)_2_ fragments. Association and dissociation were measured for 600s and 3-6 cycles of regeneration used 2.5 M guanidinium hydrochloride, 8% isopropanol between binding reactions. While theoretically a 1:1 model is not ideal for fitting bivalent IgG or F(ab’)_2_ binding data, other models did not improve curve fits and apparent binding avidities (K^app^_D_) are reported from global fits to the 1:1 model (Octet Data Analysis 10.0 software).

### Calcium Mobilization

Calcium mobilization in receptor expressing IIA1.6 cells utilized Fura-2 reporter (Abcam, Melbourne, Australia) as previously reported with the addition of probenecid (2.5 mM) to the measurement buffer (39). Loaded cells (4x10^6^/180 µl per well) were treated with 20 µl of 200, 100, or 50 µg/ml agonist mAb 8.2 or 8.26 (final volume 200 µl) and calcium flux, the ratio of A520nm obtained with 340 and 380nm excitation, was measured using a FlexStation-3 (Molecular Devices, LLC. CA USA).

### Data and Statistical Analysis

Platelet activation responses (**Figure 4D**) were fitted using Prism software (GraphPad Software, San Diego, CA), to agonist concentration *vs*. response (the bottom and top values were unconstrained), and IgG binding to FcγRIIa expressing cells used a single binding site model (**Figures 4E** and **5B, C**). Phosphorylation, binding and calcium flux activities were compared by ANOVA with Dunnett’s post comparison test and were further quantified by area under curve analysis. Data from BLI experiments used Octet Red Data Acquisition software (version 9.0.0.26, Pall ForteBio, LLC) and was fitted using data analysis software 10.0.3.1 (ForteBio, Inc).

## Results

### Agonist mAbs 8.2 and 8.26 Trigger FcγRIIa Phosphorylation and Activation in IIA1.6 Cells Expressing FcγRIIa

**Figure 1:**
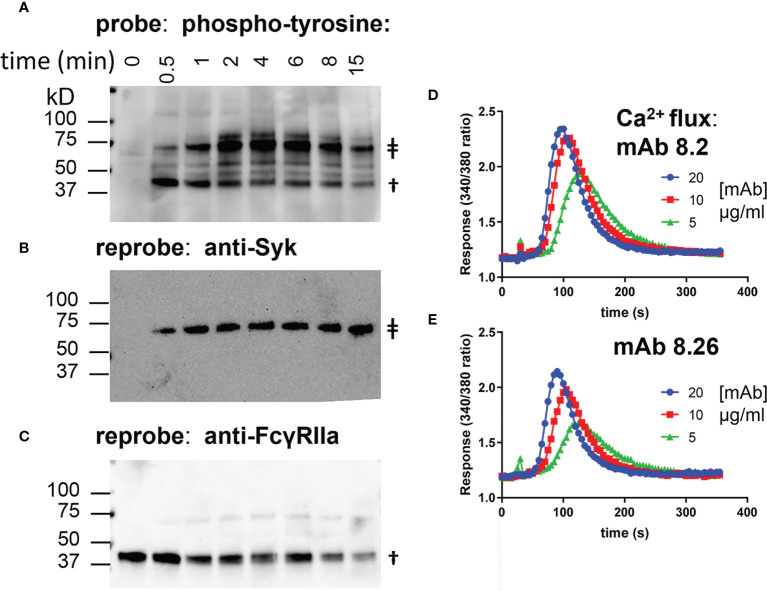
mAb 8.2 stimulates tyrosine phosphorylation of FcγRIIa-WT and mAb 8.2 and mAb 8.26 stimulate cell Ca^2+^ flux. **(A–C)** Western blots of proteins captured by the plate-bound anti-FcγRIIa mAb 8.7 from lysates of mAb 8.2 activated FcγRIIa-WT cells. Detection was, **(A)** phosphotyrosine, **(B)** Syk and **(C)** FcγRIIa. The symbol ‡ indicates syk at 72 kD and † indicates FcγRIIa at ~40 kD. **(D, E)** calcium mobilisation by IIA1.6 FcγRIIa-WT (H131) cells loaded with Fura-2 and treated with mAbs 8.2 and 8.26 as indicated.

The anti-receptor agonist mAbs 8.26 and 8.2 bind and activate the low affinity Fc-gamma receptor FcγRIIa. FcR deficient IIA1.6 cells were transduced to express FcγRIIa-H131 as an experimental system for exploring the requirements for receptor activation by these mAbs. Receptor activation was determined by detecting FcγRIIa phosphorylation in a Western blot wherein receptor and receptor associated proteins were captured by plate-bound mAb 8.7 (**Figures 1A–C**). Treatment of IIA1.6-FcγRIIa cells with agonist mAb 8.2 resulted in phosphorylation of the receptor and associated proteins (e.g., pSyk, **Figure 1B**) with similar kinetics to that found in a conventional immunoprecipitation and Western analysis (**Supplementary Figure 1**).

Calcium mobilisation is a more distal event downstream of the initial receptor phosphorylation. Both mAb 8.26 and mAb 8.2 induced concentration-dependent calcium mobilisation by FcγRIIa (**Figures 1D, E**). MAb 8.2 was the more effective agonist by both receptor phosphorylation (**Supplementary Figure 1**) and calcium flux (**Figures 1D, E**).

### Mutation of the Epitope and Fc Binding Sites of FcγRIIa

Both mAb interactions with their epitopes on FcγRIIa and the engagement of their Fc portions with the receptor may contribute to receptor activation by the mAbs 8.2 and 8.26. The contribution of each was examined using mutant receptors with abrogated mAb Fc or Fab binding. Fc binding activity of the receptor was inactivated by mutating the essential tryptophan sandwich (W87A, W110A) and Fab binding by the R55A mutation in domain 1. R55 was determined to be a key residue of the epitope for mAb 8.2 and 8.26 in a broad mutational analysis of FcγRIIa (Wines and Trist unpublished).

**Figure 2:**
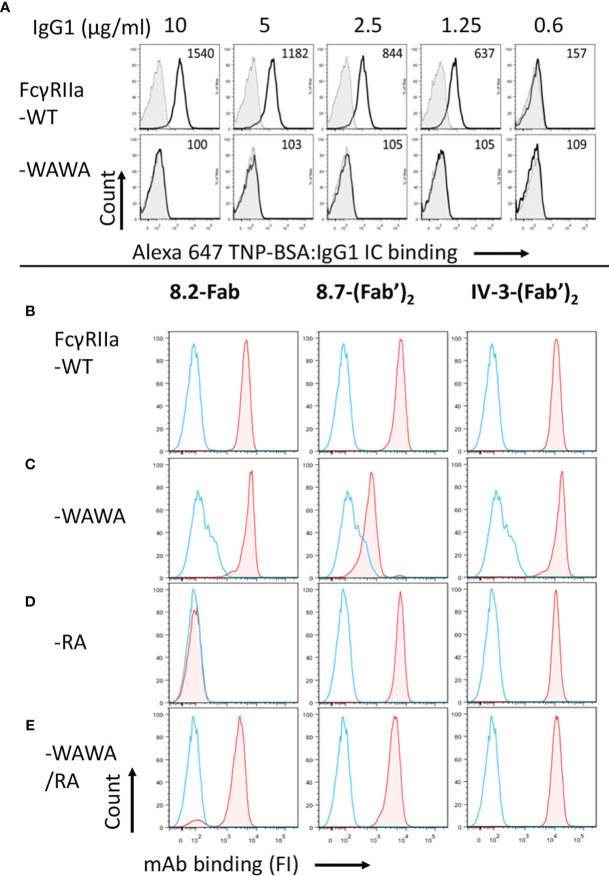
The receptor mutant FcγRIIa-WAWA does not bind ICs and FcγRIIa-RA is inactive in mAb 8.2 Fab binding. **(A)** flow cytometry analysis of IgG immune complex binding by IIA1.6 cells expressing FcγRIIa-WT (upper panel) or mutant FcγRIIa-WAWA (lower panel) inactivated for Fc binding. Cells were reacted with Alexa-647 labelled TNP-BSA:anti-TNP hIgG1 ICs at the indicated concentrations. The background binding to parental IIA1.6 cells for each concentration is shown as filled grey histograms. **(B–D)** epitope mapping/validation of mAb 8.2, by flow cytometry of anti-receptor mAb-biotin staining of IIA1.6 cells expressing **(B)** FcγRIIa-WT, **(C)** mutant FcγRIIa-WAWA, **(D)** mutant FcγRIIa-RA or **(E)** co-expressing FcγRIIa-WAWA and FcγRIIa-RA. Bound mAb was detected using streptavidin conjugated PE, blue histograms show staining with streptavidin conjugated PE alone. See **Supplementary Figure 2** for a summary of the expression data.

The inactivation of mutant FcγRIIa-WAWA (W87A, W110A) for Fc ligand binding was demonstrated by the lack of binding human IgG1 ICs (10 µg/ml IC, MFI = 100; background MFI= 109) in comparison to cells expressing the WT receptor, FcγRIIa-WT (MFI= 1540, **Figure 2A**). The anti-FcγRIIa mAbs 8.7 and IV-3 detect different distinct FcγRIIa epitopes and were used together to show the otherwise normal epitope expression by these mutant receptors and further demonstrate equivalent cell surface expression of these mutant receptors to FcγRIIa-WT (**Figure 2** and **Supplementary Figure 2**). Thus, this mutant FcγRIIa-WAWA binds the agonist mAbs 8.2 and 8.26 through their Fabs but, as expected (29), is ineffective in binding *via* the Fc. The WAWA mutation, in addition to abrogating Fc binding (**Figure 2A**), also partially disrupts the epitope of mAb 8.7 but not that of mAb IV-3 (**Figures 2B**, c.f. **C**).

Similarly, flow cytometric analysis indicated the R55A mutation in domain 1 of FcγRIIa abrogated mAb 8.2 Fab binding (**Figure 2D**), despite the overall similar expression of the mutant receptors indicated by the equivalent binding of the F(ab’)_2_ fragments of mAbs IV-3 to all mutant receptors (**Figure 2** and **Supplementary Figure 2**). The R55A mutation is distant from the Fc ligand binding site in the second ectodomain and, as expected, did not affect the binding of IgG IC (**Supplementary Figure 3**).

**Figure 3:**
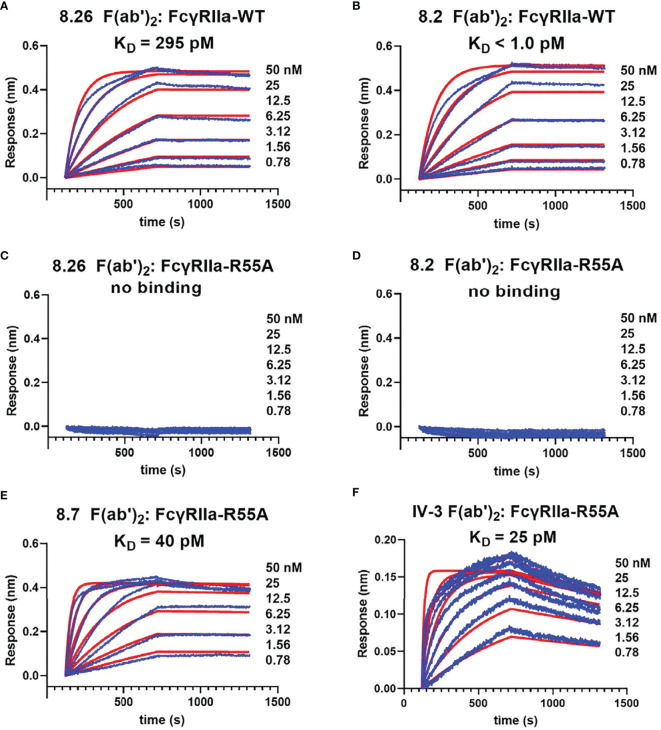
Biolayer interferometry demonstrates the mAb 8.2 and 8.26 epitope is inactivated by the mutation R55A. For biolayer interferometry rsFcγRIIa proteins **(A, B)** WT or **(C–F)** R55A mutant, were immobilized on streptavidin sensors and reacted with the indicated concentrations of mAb or their F(ab’)_2_ fragments (shown in blue). The apparent dissociation constants, K_D_^app^(nM), were derived from global fitting of the association and dissociation curves to a 1:1 Langmuir binding model (shown in red), and although a more complex (e.g. bivalent) model would theoretically be preferred, this did not provide better fitting of the data. The mAb proteins were, **(A, C)** 8.26 F(ab’)_2_, **(B, D)** 8.2 F(ab’)_2_, **(E)** 8.7 F(ab’)_2_ and **(F)** 8.2 F(ab’)_2_.

Further characterization of mutant FcγRIIa-R55A used immobilisation of the c-terminal biotinylated WT and R55A receptor ectodomains on BLI streptavidin biosensors. This format, reflects the orientation of interactions of the mAbs with the cell surface receptors with the F(ab’)_2_ fragments of the mAbs binding avidly to the immobilised FcγRIIa-WT. However, the very slow dissociation of the mAbs and the time frame of the experiments means that the dissociation rates are likely sub-optimally quantitated (42). None-the-less the apparent avidity of the F(ab’)_2_ fragment of 8.2 for FcγRIIa-WT (K^app^_D_ < 1 pM) was at least 300-fold stronger than that of the F(ab’)_2_ fragment of 8.26 (K^app^_D_ = 295 pM, c.f. **Figures 3A, B**), while binding to the mutant FcγRIIa-R55A was not detected over this same concentration range in either case (**Figures 3C, D**). The apparent avidity of the blocking mAbs 8.7 and IV-3 at 40 and 25 pM, was intermediate between that of 8.2 and 8.26 (**Figures 3E, F**).

### Functional Evaluation: Fab Interaction Is Essential for Activation

**Figure 4:**
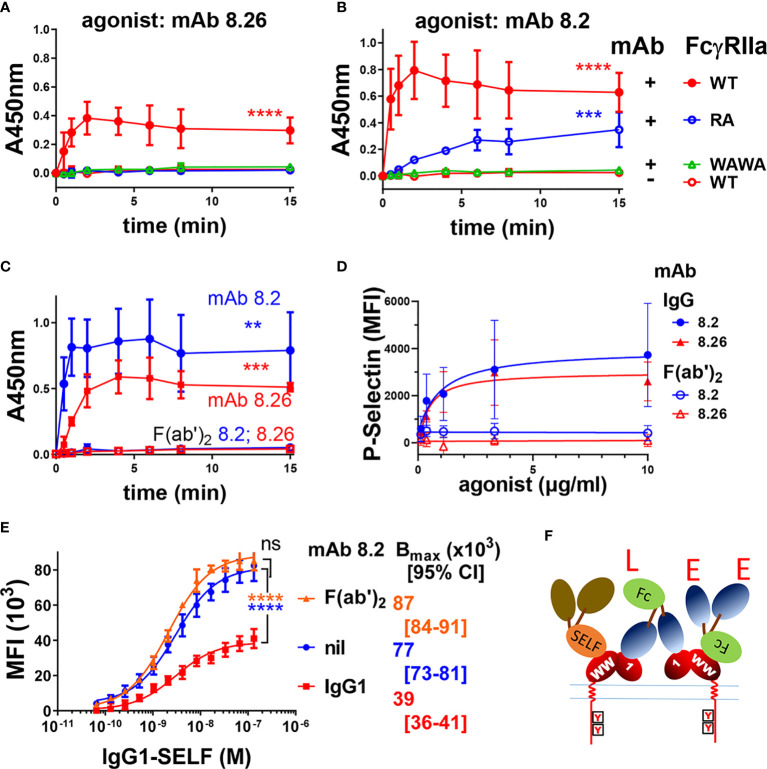
FcγRIIa activation by anti-receptor mAbs requires both Fc : FcγR binding function and an intact epitope with arginine at position 55. Intact mAbs induce receptor phosphorylation **(A, B)** of IIA1.6 cells expressing FcγRIIa-WT, the Fc-binding inactive mutant FcγRIIa-WAWA or the epitope inactive FcγRIIa-RA (R55A) as indicated, were untreated (nil mAb) or stimulated with the intact mAbs **(A)** 8.26 or **(B)** 8.2. At the indicated times cell lysates were prepared and mAb IV-3 captured phospho-proteins were detected in the FcγRIIa phosphorylation assay using with mAb 4.G10-biotin (n = 3 ± SD). **(C)** F(ab’)_2_ fragments of mAb 8.2 or 8.26 do not activate FcγRIIa. FcγRIIa phosphorylation ELISA, using FcγRIIa (mAb 8.7) capture and IIA1.6 cells expressing FcγRIIa-WT with treatment at the indicated times with either intact agonist mAb 8.2 or 8.26 (20 µg/ml) or their F(ab’)_2_. (n = 3 ± SD). **(D)** Analysis of human platelet activation using the marker P-selectin by flow cytometry following the treatment of platelet rich plasma with mAb 8.2, mAb 8.26 or their (Fab)’_2_ fragments (n = 3 ± SD, 2 individuals). Representative flow cytometry histograms are presented in **Supplementary Figure 5**. **(E)** MAb 8.2 Fc engagement competitively inhibits half of the ligand binding sites on FcγRIIa expressing cells. IIA1.6 cells expressing the R131 allelic variant FcγRIIa-R131 were untreated or treated with mAb 8.2 (n = 3 ± SD) or its F(ab’)_2_ (n = 2 ± SD) (10 µg/ml) followed by the indicated concentrations of the high affinity ligand, (anti-TNP chimeric) human IgG1-SELF. The binding of the monomeric IgG1-SELF was detected with APC-labelled anti-human IgG-F(ab’)_2_ using flow cytometry. 2-way ANOVA with Dunnett’s multiple comparison test; ns, **p < 0.0021 ***p < 0.0002; ****p < 0.0001. **(F)** A schematic representation of agonist mAb interactions with cellular FcγRIIa. The formation of larger complexes is suggested by the available ligand (‘L’ symbol) and epitope (‘E’ symbol) sites. These indicate that the Fc and Fabs of the mAbs participate in further interactions with receptors in addition to those shown. The Fc ligand ‘L’ may bind receptor on the same cell, in cis, the so-called ‘scorpion’ or Kurlander effect, or bridge to receptor on other cells in trans. The blocking of half the receptor ligand binding sites with saturating mAb binding **(E)** suggests most interactions are in cis. This is indicated by the high affinity binding of IgG1-SELF *via* its Fc to only one of the two receptors. The first domain (label 1) of the receptor ectodomain contains the mAb 8.2/8.26 epitope (R55), and the “WW” on EC2 indicates the conserved tryptophans essential to FcγR binding to Fc.

IIA1.6 cells expressing FcγRIIa-WT or FcγRIIa-RA were treated with the mAbs and receptor activation evaluated in a FcγRIIa phosphorylation ELISA. These assays used capture of receptor from the cell lysates by plate bound mAb IV-3 (or 8.7) rather than immunoprecipitation (**Figures 1A–C** and **Supplementary Figure 1**). While intact mAb 8.26 treatment of FcγRIIa-WT expressing cells developed a strong pTyr signal peaking at 2 to 4 min, FcγRIIa-RA cells induced no receptor phosphorylation (**Figure 4A**) consistent with the lack of its F(ab’)_2_ fragment binding to rsFcγRIIa-RA (**Figure 3C**). In contrast despite the lack of mAb 8.2 F(ab’)_2_ fragment binding to rsFcγRIIa-RA (**Figure 3D**) the intact mAb 8.2 induced poor but detectable phosphorylation of FcγRIIa-RA (**Figure 4B**) which was much reduced compared with FcγRIIa-WT, had slower kinetics and never reached the peak pY levels that FcγRIIa-WT achieved after ~ 2 min stimulation (**Figure 4B**). BLI was performed to test for the presence of aggregates in the mAb 8.2 IgG. Size exclusion chromatography (SEC) resolved mAb 8.2 as a symmetrical peak and the leading and trailing fractions (**Supplementary Figures 4A, B**) were analyzed using rsFcγRIIa-RA immobilized sensors. In contrast to the lack of binding of the 8.2 F(ab’)_2_ fragment to rsFcγRIIa-RA (**Figure 3D**) the intact 8.2 bound strongly (**Supplementary Figures 4C, D**). If present, preformed aggregates would occur in the leading fractions of the SEC peak, however the binding activity was largely equivalent in the leading (**Supplementary Figure 4C**) and trailing (**Supplementary Figure 4D**) peak fractions. Hence preformed aggregates of mAb 8.2 do not account for the residual binding and so receptor stimulating activity of mAb 8.2 with FcγRIIa-RA. Notably the intact mAb 8.26 had comparatively little binding activity with FcγRIIa-RA (**Supplementary Figure 4E**) and a higher concentration series of an isotype control IgG1, as expected, showed micromolar binding affinity (**Supplementary Figure 4F**). Thus, as expected the Fab:epitope interaction, as defined using the RA mutation, is essential for the activating function of mAb 8.26. This interaction with R55 was required for the optimal binding and stimulating activity of mAb 8.2, but nonetheless was not essential.

### Functional Evaluation: Fc Interaction Is Essential

When IIA1.6 cells expressing Fc binding-inactive FcγRIIa-WAWA were treated with mAb 8.26 or mAb 8.2 no receptor associated pTyr was detected over the background levels measured in mock treated FcγRIIa-WT cells (**Figures 4A, B**). This result was extended by the treatment of IIA1.6 cells expressing FcγRIIa-H131 with mAb 8.2 F(ab’)_2_ fragments or 8.26 F(ab’)_2_ fragments, which similarly resulted in no receptor associated phosphorylation above background, contrasting with the activity of the intact antibodies (8.2 IgG c.f. F(ab’)_2_ p = 0.005; 8.26 IgG c.f. F(ab’)_2_ p = 0.0007) (**Figure 4C**). Thus, engaging up to two FcγRIIa-WAWA molecules by intact mAb 8.2 or 8.26, or similarly engaging two FcγRIIa-WT by their F(ab’)_2_ fragments is insufficient for receptor activation. This indicates an essential role of the mAb Fc portion in activation of the cellular receptor by these anti-FcγRIIa mAbs. Next the role of the Fc in agonism was examined on human platelets which endogenously express only FcγRIIa (18). Treatment of platelets with the mAbs 8.26 or 8.2, but importantly not their (Fab)’_2_ fragments, lead to platelet activation as measured by the increased surface expression of P-selectin (**Supplementary Figure 5**), with an EC_50_ of ~0.6 µg/ml. (**Figure 4D**). These data from IIA1.6 cells expressing FcγRIIa and from platelets together reveal that the Fc portions of these mAbs are essential for agonism of FcγRIIa in systems with both ectopically and natively expressed FcγRIIa.

The Fc can bind to a receptor on the same cell as the Fab portion(s) bind receptor(s), a cis interaction, or the Fc can bridge to a receptor on a different cell, a trans interaction. The nature of the Fc interaction within the mAb 8.2: FcγRIIA complex formed on the cell was investigated using a competitive binding assay with the mAb 8.2 or its F(ab’)_2_ fragment and human IgG1-S267E, L328F (IgG1-SELF), a monomeric high affinity Fc ligand of the R131 allele of FcγRIIa (37). In the presence of mAb 8.2 the capacity of IgG1 SELF ligand binding to FcγRIIa-R131 (B_max_) was reduced ~ 2-fold from MFI (x10^3^) = 77 to 39 (p < 0.0001) (**Figure 4E**). In contrast the F(ab’)_2_ had little effect, trending towards increase, on the number of ligand binding sites on the cell (B_max_, MFI (x10^3^) = 87, p = ns). The decreased number of ligand binding sites with bound intact mAb 8.2 is consistent with the Fc portion binding FcγRIIa and competitively inhibiting binding of the high affinity ligand IgG1-SELF. Thus, each mAb molecule binds to FcγRIIa by its two non-blocking Fabs, with its Fc also engaging a receptor, and the halving of the number of available IgG1-SELF ligand binding sites on the mAb 8.2 treated cells (**Figure 4E**). These interactions with cellular FcγRIIa are shown schematically in (**Figure 4F**) indicating each agonist mAb can participate in three possible stable interactions with receptors (2x Fab + 1x Fc), which at binding saturation of all the cellular receptors results in the Fc interactions occupying half the ligand binding sites (FcγRIIa) of the cell. This also suggests that such tripartite binding is predominately a cis interaction on the cell surface and may be a requirement for the FcR activation by these mAbs. This could not be achieved by a binding dominated by trans interactions which are limited to receptors at points of contact between cells. Since the Fab and Fc interactions are non-competitive, crosslinking of receptors into large complexes is likely (note unoccupied ligand binding sites ‘L’ and epitopes ‘E’ in **Figure 4F**).

### Fab and Fc Interaction With FcγRIIa Must Occur on the Same Receptor Molecule for mAb-Stimulated Receptor Activation

**Figure 5:**
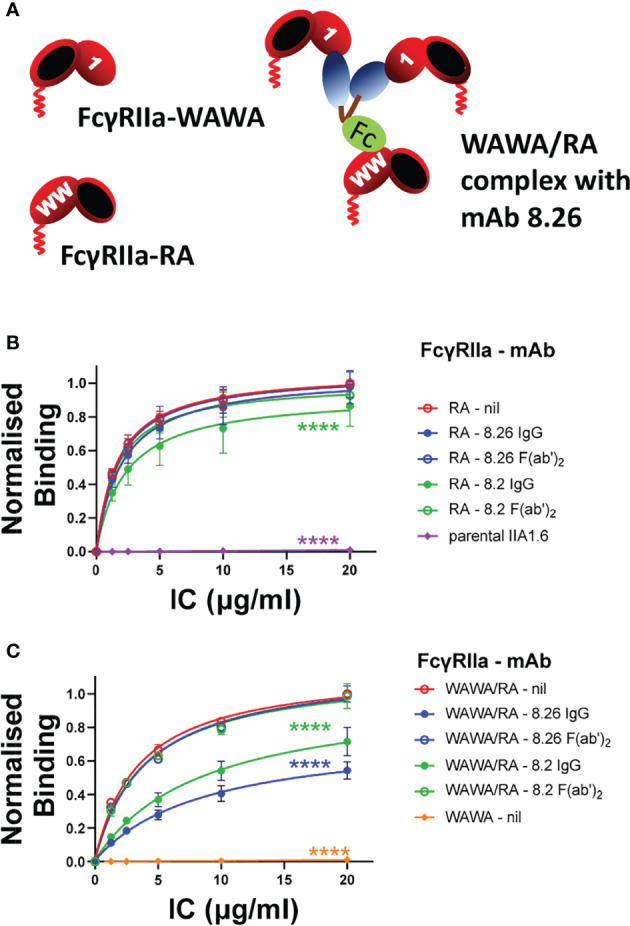
**(A)** Schematic diagram of the interaction of the mAbs 8.2 or 8.26 with FcγRIIa-WAWA (inactive in binding the Fc ligand) and with co-expressed FcγRIIa-RA (R55A mutation destroys the mAb’s epitope). The Fab and Fc of mAbs interact with FcγRIIa-WAWA and FcγRIIa-RA respectively when co-expressed in the same cell. **(B, C)** IgG IC binding to FcγRIIa-WAWA/FcγRIIa-RA co-expressing cells is competitively inhibited by the intact mAb 8.26 or 8.2 but not their F(ab’)_2_ fragments. The binding of IC, chemically crosslinked and biotinylated human IgG, was performed in the absence (nil) or presence of (10 µg/ml) mAb 8.2 or 8.26 IgG or their F(ab’)_2_ fragments as indicated and was measured by incubation with APC-labelled streptavidin by flow cytometry (**Supplementary Figure 6**). IC binding was to **(B)** FcγRIIa-RA cells, or parental IIA1.6 cells, and **(C)** FcγRIIa-WAWA/FcγRIIa-RA co-expressing cells, or FcγRIIa-WAWA cells. Normalized geometric mean ± SD, n = 3. 2-way ANOVA with Dunnett’s multiple comparison test to untreated IC binding; ****p < 0.0001.

Since FcγRIIa stimulation by the mAbs requires participation of both the Fab and Fc portions and since the intact mAbs exhibit tripartite F(ab’)_2_ + Fc binding we tested whether mAb stimulated receptor activation would still occur if these interactions were segregated onto different receptor molecules (**Figure 5A**). Fab and Fc binding was segregated to FcγRIIa-WAWA and FcγRIIa-RA co-expressed in the same cell. Staining of these co-expressing cells with mAb 8.2 (Fab) and mAb 8.7 confirmed the simultaneous expression of FcγRIIa-WAWA and FcγRIIa-RA (**Figure 2E**). While FcγRIIa-WAWA exclusively binds the mAbs 8.2 and 8.26 F(ab’)_2_ fragments, a competitive IC binding assay was used to confirm the participation of the mAb Fc in binding to the FcγRIIa-RA on these co-expressing cells (**Supplementary Figure 6**). As expected, IgG immune complex binding to FcγRIIa-RA expressing cells was not inhibited by mAb 8.26, either as intact IgG or as F(ab’)_2_ fragments, since binding is abrogated to the disrupted epitope (**Figure 5B**). A small inhibition of IC binding was observed with intact mAb 8.2 IgG (**Figure 5B**), again suggesting this mAb retains some interaction with the RA mutant receptor. As expected, there is no IC binding to the FcγRIIa-WAWA cells (**Figure 5C**), but IC binding occurs as expected to the co-expressed FcγRIIa-RA/FcγRIIa-WAWA. Notably the intact IgG form of the mAbs 8.26 and 8.2 markedly inhibit IC binding (****, p < 0.0001), while their F(ab’)_2_ fragments are ineffectual. Thus, in these cells, when the agonist mAb binds the unaltered epitope of FcγRIIa-WAWA molecules *via* its two Fabs, its Fc can also bind co-expressed FcγRIIa-RA (**Figure 5A**) and so inhibit IC binding (**Figure 5C**).

**Figure 6:**
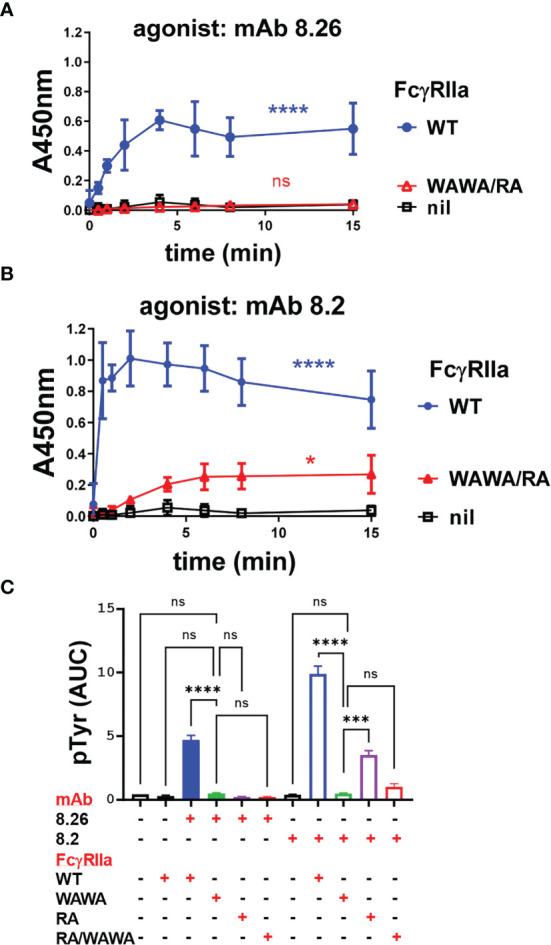
**(A, B)** MAb 8.26 or 8.2 induced FcγRIIa tyrosine phosphorylation in IIA1.6 cells expressing wild type FcγRIIa-WT, or co-expressing FcγRIIa-WAWA and FcγRIIa-RA or parental cells lacking FcγR. Tyrosine phosphorylation was measured in the FcγRIIa phosphorylation ELISA, n = 3. 2-way ANOVA with Dunnett’s multiple comparison test to untreated WT; ns-not significant, *p < 0.0332, ****p < 0.0001. **(C)** Area under curve analysis of the receptor activation in **Figures 4A, B** and **Figures 6B, C**. One-way ANOVA with Dunnett’s multiple comparison test showing comparison to FcγRIIa-WAWA ***p 0.0003, ****p < 0.0001.

Having demonstrated Fab binding to FcγRIIa-WAWA and Fc binding to co-expressed FcγRIIa-RA the ability of mAb to activate these complexes was determined. MAb 8.26 did not elicit receptor-associated phosphorylation above background in the FcγRIIa-WAWA/FcγRIIa-RA mutant co-expressing cells (**Figure 6A**). Furthermore, mAb 8.2 induced only weak receptor-associated phosphorylation in the FcγRIIa-WAWA/FcγRIIa-RA co-expressing cells (**Figure 6B**) which was similar to that observed in the FcγRIIa-RA cells (**Figures 4B**, **6C**). This weak receptor-activation was similar, both in terms of the reduced levels of phosphorylation and in its delayed kinetics (**Figure 4B** cf. **Figures 6B, C**). The poor but detectable phosphorylation induced by mAb 8.2, is again attributed to the residual binding to the FcγRIIa-R55A, as observed in the BLI analysis (**Supplementary Figure 4**). Thus, co-expression of the FcγRIIa-WAWA/FcγRIIa-RA mutants did not result in rescue of anti-FcγRIIa mAb receptor phosphorylation since the mAb 8.2 induced a similar weak receptor-activation in the cells expressing FcγRIIa-RA alone.

**Figure 7:**
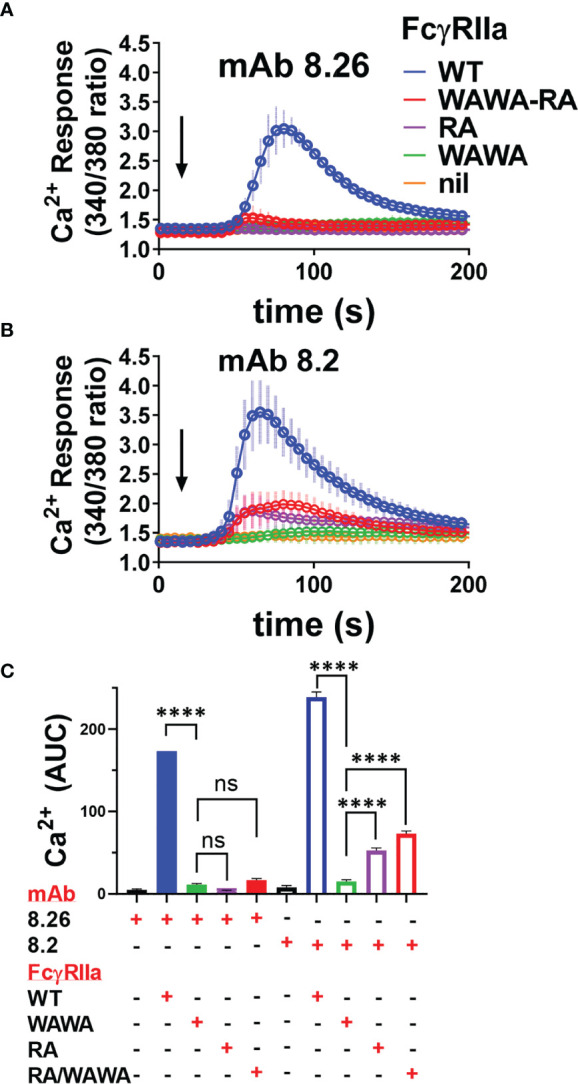
Calcium mobilisation in mAb treated FcγRIIa expressing cells is not effectively rescued in FcγRIIa-WAWA/FcγRIIa-RA co-expressing cells. Fura-2 loaded IIA1.6 cells lacking receptor (nil), or expressing FcγRIIa-WT, FcγRIIa-WAWA, FcγRIIa-RA or co-expressing FcγRIIa-WAWA and FcγRIIa-RA (WAWA-RA) were treated with **(A)** agonist mAb 8.26, 20 µg/ml (n = 5, 340/380nm FI ± SEM) or **(B)** 8.2, 10 µg/ml (n = 3-5, ± SEM). **(C)** Area under curve analysis of Ca^2+^ mobilisation in **(A, B)** with 1-way ANOVA with Dunnett’s multiple comparison test showing comparison to FcγRIIa-WAWA, ****p < 0.0001.

Lastly, mAb activation of FcγRIIa-WT and FcγRIIa mutant receptor expressing cells was assessed by calcium mobilisation, as a sensitive measure of events distal to receptor phosphorylation. The two agonist mAbs induced robust calcium flux in FcγRIIa-WT expressing cells but, consistent with the phosphorylation studies, neither mAb 8.26 nor 8.2 induced calcium mobilisation in FcγRIIa-WAWA cells, confirming the necessity for Fc engagement for the mAb activity (**Figures 7A, B**). MAb 8.26 treatment showed negligible activation of either FcγRIIa-RA expressing or FcγRIIa-WAWA/FcγRIIa-RA co-expressing cells (**Figure 7A**). Thus, the complex formed on the FcγRIIa-WAWA/FcγRIIa-RA co-expressing cells, comprising of one-mAb engaged in up to three interactions with receptors (2x Fab + 1xFc), was ineffective in receptor activation. Furthermore, Fc and Fab binding to the same FcγRIIa molecule is required for the mAb 8.26 stimulated receptor activation.

MAb 8.2 induced calcium fluxes that were similarly reduced in peak height and area in both the FcγRIIa-RA single expressing and FcγRIIa-WAWA/FcγRIIa-RA co-expressing cells (**Figures 7B, C**). These equivalent muted calcium fluxes, mirror the reduced levels of receptor phosphorylation, and together indicate that, for this mAb also, optimal activation is not restored upon co-expression of the two mutant receptors (i.e., FcγRIIa-WAWA with FcγRIIa-RA).

## Discussion

The recognition by FcγRs of IgG complexed with antigen, activates receptor bearing cells and so specifically directs innate effector cells to targets of adaptive humoral immunity (2–7, 43). In this study we explored FcγR activation by two agonistic and non-blocking anti-FcγRIIa mAbs, mAb 8.26 and mAb 8.2. BLI analysis revealed high apparent binding avidities for the bivalent F(ab’)_2_ fragments, with apparent avidities of ~ 300 pM for the weaker activating mAb 8.26 and < 1 pM for the stronger activator mAb 8.2. The epitopes of both mAb 8.26 and mAb 8.2 were disrupted by the R55A mutation in the first extracellular domain, ablating binding of the F(ab’)_2_ fragments of both 8.26 and 8.2, but leaving residual Fc-dependent binding of the intact mAb 8.2. The activation of FcγRIIa-RA was completely abrogated for mAb 8.26, and largely abrogated for mAb 8.2. For mAb 8.2 residual activation was consistent with the residual binding activity of this intact mAb for the epitope mutated receptor. This binding of mAb 8.2 to FcγRIIa-RA indicates its recognition of FcγRIIa differs from mAb 8.26, despite R55 being a key feature of the epitopes of both mAbs.

Receptor activation by both mAb 8.26 and 8.2 depends upon Fab binding to epitopes that do not inhibit the ligand binding function of the receptor, thus allowing their Fcs to also bind FcγRIIa. Indeed, receptor activation by these mAbs required the mAb-Fc to interact with the receptor since their F(ab’)_2_ fragments did not activate cells, and cells expressing the mutant FcγRIIa-WAWA, which is inactive in Fc binding, was not activated by either intact mAb 8.26 or 8.2. Thus, the binding of two receptors, two WT receptors by F(ab’)_2_ fragment or two FcγRIIa-WAWA mutant receptors by intact mAb, is insufficient for signalling.

Furthermore, this failure of the intact mAbs to elicit functional activity from FcγRIIa-WAWA indicated that Fc : Fc interactions, that promote the ordered aggregation of antibodies in antigen:IgG complexes (44, 45) are, not sufficient for activation by these mAbs. That is, if Fc : Fc interactions do occur, in addition to Fab : FcγRIIa interactions with these mAbs, these are insufficient for receptor activation. Rather Fc : FcγRIIa and Fab : FcγRIIa interactions are required for activation.

Having shown that receptor activation by these agonist mAbs required both Fab and Fc interaction we tested if these interactions would still stimulate receptor activation when segregated to separate receptor molecules. The co-expression of mutant receptors was used to test this engagement of separate receptors with only Fab and only Fc binding activity. FcγRIIa-RA lacks mAb 8.26 and 8.2 F(ab’)_2_ fragment binding but binds the Fc normally. However, co-expression of this FcγRIIa-RA with FcγRIIa-WAWA, lacking Fc binding activity, was not permissive of receptor activation by the mAb 8.26. The mAb 8.26 : FcγRIIa-RA : FcγRIIa-WAWA-RA : FcγRIIa-WAWA complex formed on these cells will comprise one-mAb contributing up to three interactions with receptors (2x Fab + 1x Fc), but this was ineffective in receptor activation. The requirement for Fab and Fc interactions on the same FcγRIIa molecule indicates that further crosslinking of receptors is necessary for activation. MAb 8.2 mediated stimulation was similarly not rescued with the co-expression of FcγRIIa-RA/FcγRIIa-WAWA receptors. The similarly muted calcium fluxes and receptor phosphorylation seen when FcγRIIa-RA alone was treated with 8.2, suggests mAb 8.2 and 8.26 differ in some aspect of their manner of receptor binding and stimulation.

The key role of the FcR : Fc interaction in activation by the mAbs is notable as the mAb 8.2 is a mouse IgG1 subclass which is a poor Fc ligand of the human FcγRIIa-His131 allelic form used in the receptor activation studies herein (15, 16). Intact mAb is likely to avidly bind by its Fabs (K^app^_D_ ~ pM for F(ab’)_2_ by BLI) to receptors on the cell surface and so co-localise and present the Fc portion for binding in cis. Indeed, cell binding analysis (**Figure 4E**) showed the mAb 8.2 presented Fc engages with receptor and, despite this interaction only having micromolar intrinsic affinity (9), does so sufficiently strongly to inhibit the binding of a high affinity mutant human IgG1 [S267E, L328F, K_D_ of ~ 4 nM (37)] to half the receptors (FcγRIIa-R131) of the cell. Although some trans mAb interactions bridging between cells may occur, the occupation of half the ligand binding sites on the mAb saturated cells appears consistent with largely cis interactions on the same cell. In this way, even intrinsically low affinity interactions are favoured by the apparent high local concentration of ligand, in this case the co-localised Fc of the agonistic mAbs. This phenomenon, known as the “scorpion” or Kurlander effect (6, 46), can enhance apparent interaction affinities by many orders of magnitude (47). Thus, the low intrinsic affinity of mouse IgG1-Fc interaction with FcγRIIa-His131 does not preclude its critical importance in the activation of FcγRIIa expressing cells by mAb 8.2.

More broadly this shows a Fc : FcR interaction that in itself is normally too weak to bind appreciably can be sufficient to result in Fc : FcR interaction, when appropriately co-localised to the cell surface by Fab binding, and can thus initiate cell activation and biological responses. This has important implications for the possibility of low affinity FcR-dependent mechanisms of action of other antibodies that target cells bearing FcγRs and have low, but not completely abrogated, FcγR binding activity. Unforeseen functional responses dependent on very weak FcγRII interactions have triggered adverse reactions to therapeutic mAbs under other circumstances (48) or confounded mAb therapy by modulation of the target molecule (49–51).

Other studies are compatible with the conclusion herein that small immune complexes are unable to activate FcγRs. Recombinant trimeric human IgG-Fc molecules have been developed recently which effectively bind, but do not activate FcγRs, and so are inhibitors of FcγR function. These molecules had therapeutic efficacy in several mouse models of autoimmune antibody mediated pathology (52). In other studies, engineered IgGs comprising two or three Fc regions were not activating in themselves but had avidity enhanced potency over normal IgG when aggregated on a target (53–55). Recent imaging studies found a pentameric Fc-ligand recruited and activated Syk to the endosomes of FcγRI^+^, FcγRIIa^+^ THP1 cells. In contrast, activation by large complexes occurred at the plasma membrane (56). In summary, our study found activation of FcγRIIa by the non-blocking agonist mAbs 8.26 and 8.2 required interactions with the Fab and the Fc on the same receptor molecule, which enables signalling permissive receptor crosslinking. MAb interactions limited to only two (e.g., F(ab’)_2_ fragment) or three receptors did not lead to receptor activation. These findings, applied more generally, will assist in the development of more ‘tuneable’ therapeutic mAbs.

## Data Availability Statement

The raw data supporting the conclusions of this article will be made available by the authors.

## Ethics Statement

The studies involving human participants were reviewed and approved by Alfred Hospital Ethics Committee. The patients/participants provided their written informed consent to participate in this study.

## Author Contributions

BW and PH conceived and designed the experiments. BW, PH, GM, HT, SE, RI, TC, RA, and RB designed assays. BW, HT, SE, RI, GP, and TC performed experimental work and analyzed results. BW, PH, TC, and GM drafted the manuscript. All authors contributed to the article and approved the submitted version.

## Funding

This work was supported by the Australian NHRMC – Project Grant, to PH and BW (GNT1145303). Support to the Burnet Institute comes from the NHMRC Independent Research Institutes Infrastructure Support Scheme and a Victorian State Government Operational Infrastructure grant. TC holds an Australian Research Council fellowship (DE190100806).

## Conflict of Interest

The authors declare that the research was conducted in the absence of any commercial or financial relationships that could be construed as a potential conflict of interest.

## Publisher’s Note

All claims expressed in this article are solely those of the authors and do not necessarily represent those of their affiliated organizations, or those of the publisher, the editors and the reviewers. Any product that may be evaluated in this article, or claim that may be made by its manufacturer, is not guaranteed or endorsed by the publisher.
