# Supplementary material for: Fc Binding by FcγRIIa Is Essential for Cellular Activation by the Anti-FcγRIIa mAbs 8.26 and 8.2
Source: Front Immunol. 2021 Oct 25;12:666813. doi: 10.3389/fimmu.2021.666813 (PMC8573391; doi:10.3389/fimmu.2021.666813)
Supplement: Supplementary file 1 [file DataSheet_1.docx]

Supplementary Material

## Supplementary Figures


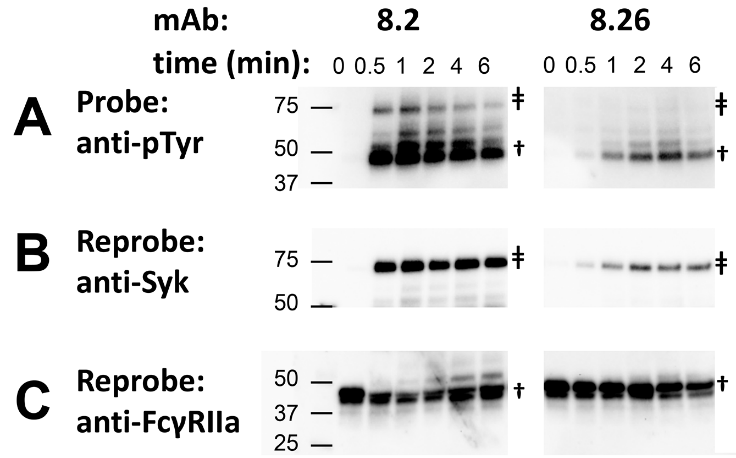


**Supplementary Figure 1**. The FcγRIIa specific mAbs 8.2 or mAb 8.26 induce phosphorylation in IIA1.6 cells expressing FcγRIIa.

**A-C)** IIA1.6 cells expressing FcγRIIa-WT (H131 allele, 4x10^6^/40 µl per time point) were treated with 20 µl of 10 µg/ml agonist mAb 8.2 or 8.26 (final volume 60 µl) and 200 µl of lysis buffer was added at the indicated times. The clarified lysate was incubated with mAb 8.7 F(ab’)_2_ fragment coupled Sepharose 4B to immunoprecipitate FcγRIIa and its associated proteins. The panels show **A)** Western blot membrane probed with mAb 4G10-biotin/streptavidin-HRP detecting phosphotyrosines, **B)** reprobed with anti-Syk and lastly, **C)** reprobed with anti-FcγRIIa. The ǂ symbol indicates syk at 72 kD and † FcγRIIa at ~40 kD


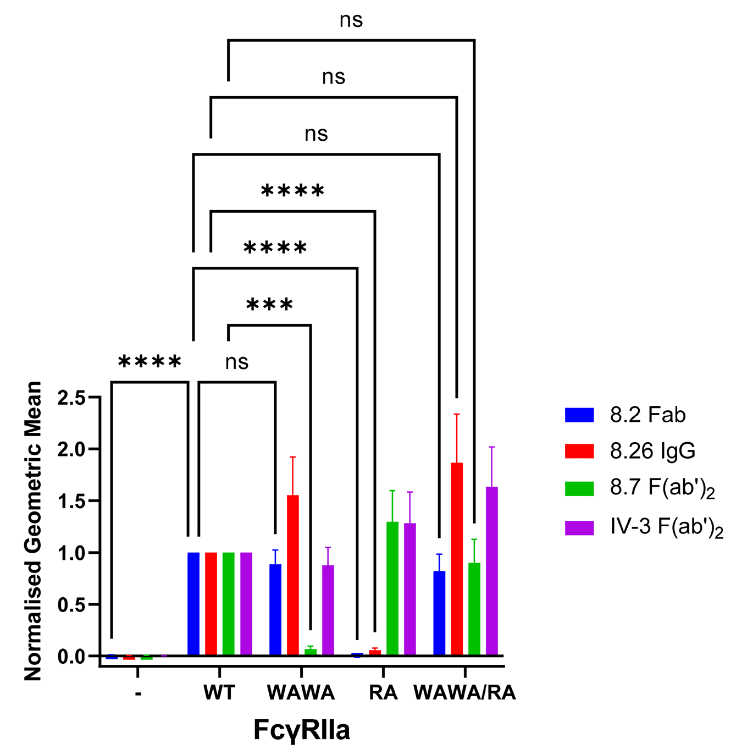

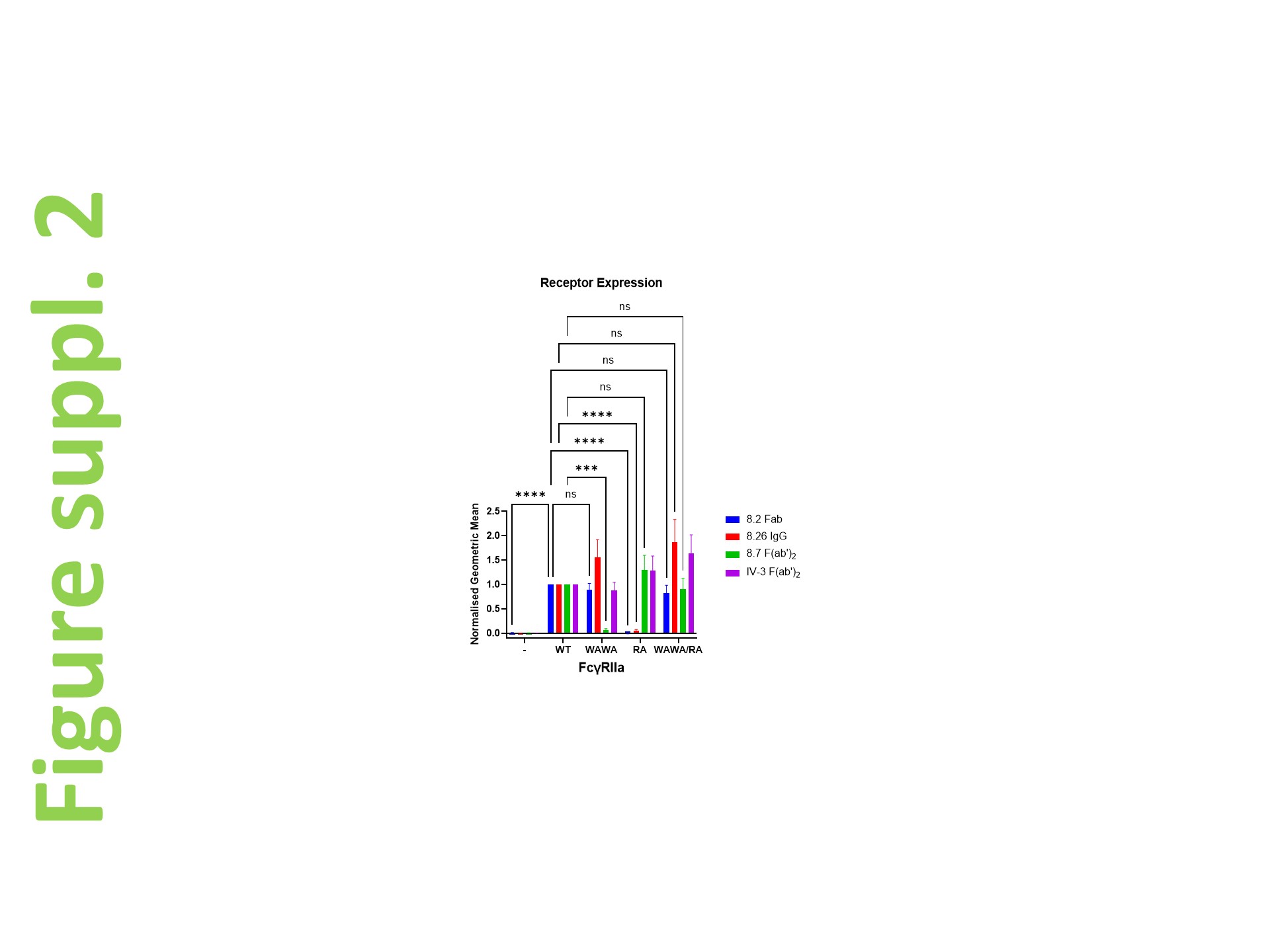


**Supplementary Figure 2**. The FcγRIIa expression on cell lines was established with the specific biotinylated mAbs or fragments, 8.2-Fab, mAb 8.26, 8.7 F(ab’)_2_ and IV-3 8.7 F(ab’)_2_ by flow cytometry. The expression (geometric mean) was normalized to that of the FcγRIIa-WT cell line (± SEM, n = 4), 2-way ANOVA, Dunnett’s multiple comparison test to FcγRIIa-WT, *** p = 0.0002, **** p < 0.0001. Some comparisons that were non-significant (ns) and some comparisons to the parental cell line (-), which were all ****, are omitted for clarity.


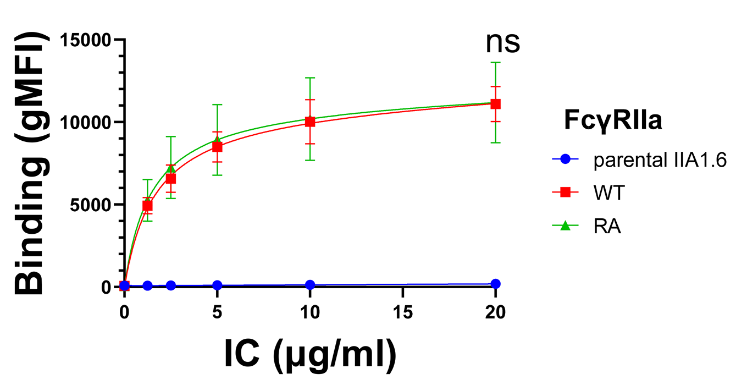


**Supplementary Figure 3.** The R55A mutant FcγRIIa (FcγRIIa-RA) is competent in immune complex ligand binding.

Biotinylated IgG-IC at 20, 10, 5, 2.5, 1.25, and 0 µg/ml were bound to FcγRIIa-WT or FcγRIIa-RA expressing cells and detected using streptavidin-APC by flow cytometry. Binding (Geometric MFI ± SD, n=3) was equivalent to the FcγRIIa-WT or FcγRIIa-RA expressing cells (p > 0.1234, ns, 2-way ANOVA with Dunnett’s multiple comparison test).


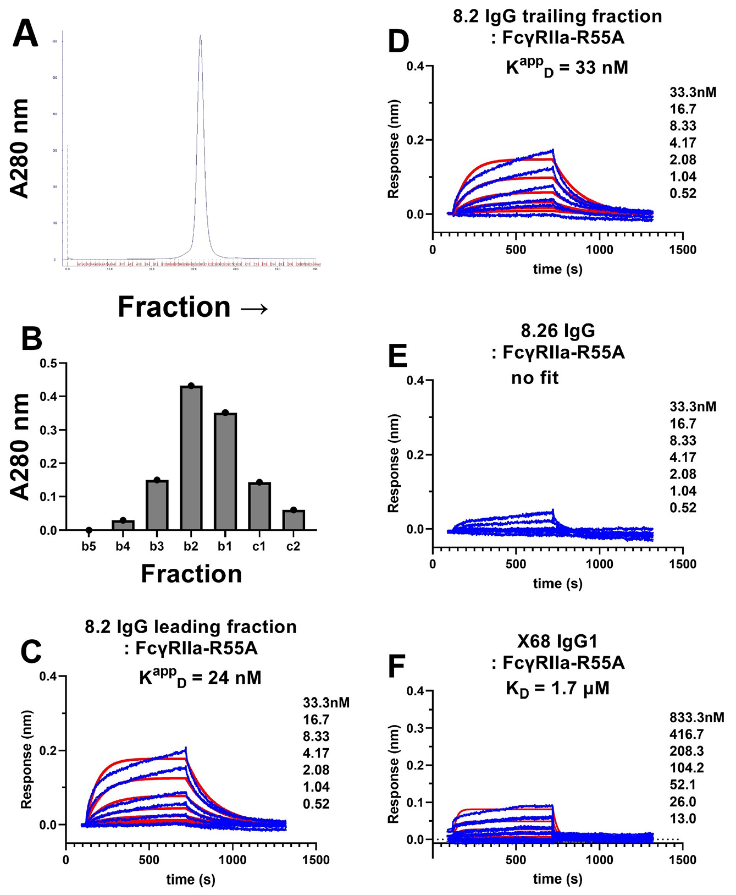


**Supplementary Figure 4.** Biolayer interferometry of SEC fractions indicates aggregates do not account for the binding of intact mAb 8.2 IgG to FcγRIIa R55A.

**A)** MAb 8.2 was resolved by SEC on a Superose 6 column as a symmetrical peak and **B)** absorbance (A280 nm) of 0.5 ml collected fractions was measured. **C)** The leading fraction b3 and **D)** the trailing fraction c1 of the mAb 8.2 peak, **E)** mAb 8.26 (a pooled SEC peak) and **F)** IgG1 isotype control mAb X68 were analyzed by BLI. RsFcγRIIa-RA was immobilized on streptavidin sensors and reacted with a concentration series (C-E, 33 to 0.5 nM, F, 833 to 13 nM) of mAb using the Octet96. Fitting (red) of data (blue) is to a 1:1 model for C, D, and F.


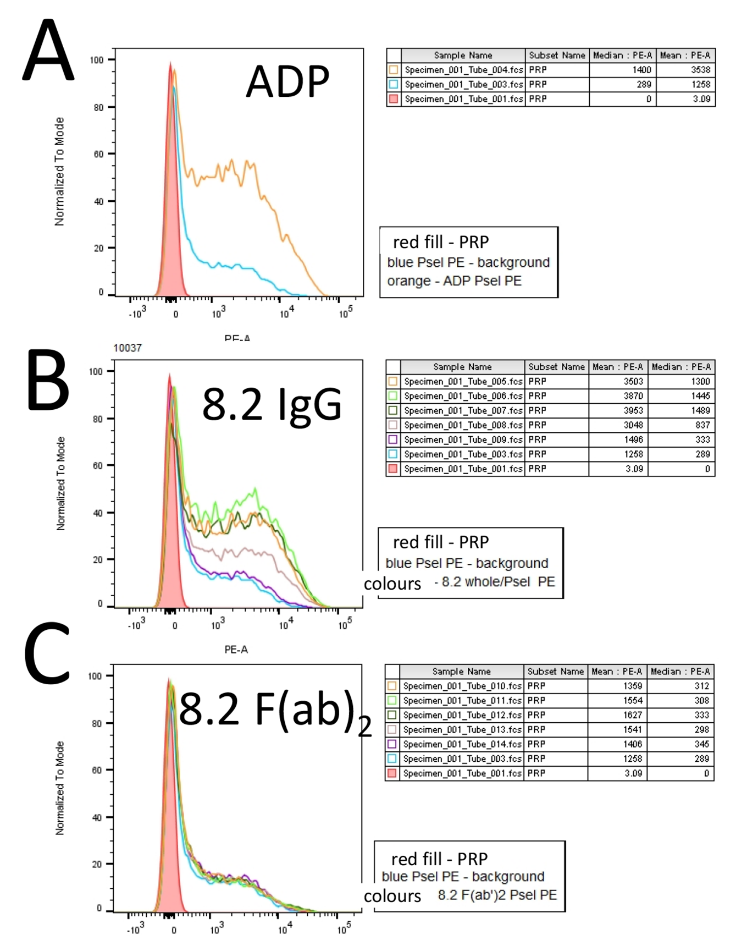


**Supplementary Figure 5.** **Platelet Activation Assay.**

Platelet activation was performed by incubating 20 µl Platelet rich plasma (PRP) with 100 µl of **A)** ADP (20 µM; 10min) or mAb 8.2, **B)** whole or **C)** F(ab’)_2_ for 30 min. Activation was determined using mouse anti-P-selectin (AK-4) phycoerythrin-conjugate by flow cytometry.


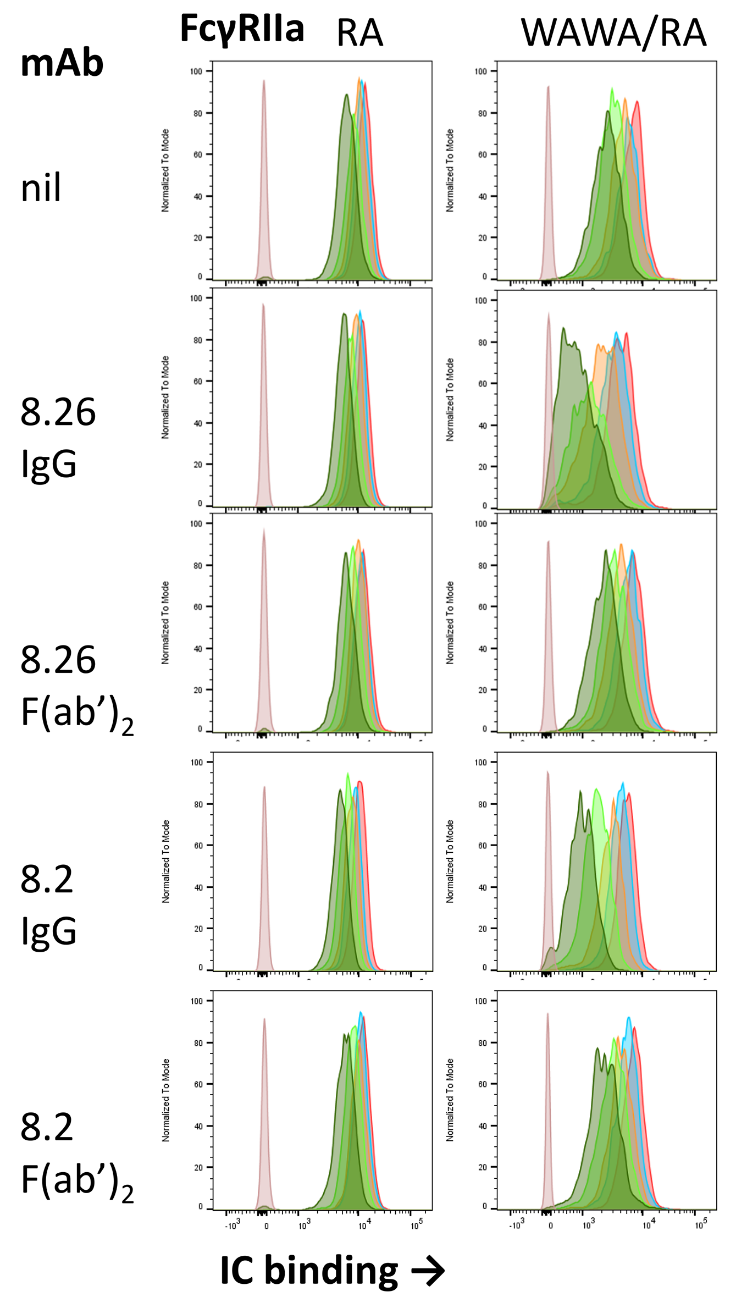


**Supplementary Figure 6.** Immune complex binding to FcγRIIa-RA/FcγRIIa-WAWA is inhibited by mAb IgG.

Biotinylated IgG-IC at 20, 10, 5, 2.5, 1.25, and 0 µg/ml were bound to FcγRIIa-RA or FcγRIIa-RA/FcγRIIa-WAWA expressing cells in the absence or presence of mAbs or their fragments and detected using streptavidin-APC by flow cytometry. Geometric means from these histograms contribute 1 representative experiment to the 3 experiments (n=3) summarized in Figure 5.
